# The A’-helix of CYP11A1 remodels mitochondrial cristae

**DOI:** 10.1186/s12929-022-00846-7

**Published:** 2022-08-18

**Authors:** Karen G. Rosal, Wei-Yi Chen, Bon-chu Chung

**Affiliations:** 1 Molecular Cell Biology, Taiwan International Graduate Program, Academia Sinica and Graduate Institute of Life Science, National Defense Medical Center, Taipei, 115 Taiwan; 2 Institute of Molecular Biology, Academia Sinica, Taipei, 115 Taiwan; 3 Institute of Biochemistry and Molecular Biology, National Yang Ming Chiao Tung University, Taipei, 112 Taiwan; 4 Graduate Institute of Biomedical Sciences, Neuroscience and Brain Disease Center, China Medical University, Taichung, 404 Taiwan

**Keywords:** Steroidogenesis, Pregnenolone, P450scc, Hsp60, Membrane, MIC10, Mitochondrial structure, Cristae remodeling

## Abstract

**Background:**

CYP11A1 is a protein located in the inner membrane of mitochondria catalyzing the first step of steroid synthesis. As a marker gene for steroid-producing cells, the abundance of CYP11A1 characterizes the extent of steroidogenic cell differentiation. Besides, the mitochondria of fully differentiated steroidogenic cells are specialized with tubulovesicular cristae. The participation of CYP11A1 in the change of mitochondrial structure and the differentiation of steroid-producing cells, however, has not been investigated.

**Methods:**

We engineered nonsteroidogenic monkey kidney COS1 cells to express CYP11A1 upon doxycycline induction and examined the mitochondrial structure of these cells. We also mapped the CYP11A1 domains that confer structural changes of mitochondria. We searched for CYP11A1-interacting proteins and investigated the role of this interacting protein in shaping mitochondrial structure. Finally, we examined the effect of CYP11A1 overexpression on the amount of mitochondrial contact site and cristae organizing system.

**Results:**

We found that CYP11A1 overexpression led to the formation of tubulovesicular cristae in mitochondria. We also identified the A’-helix located at amino acid #57–68 to be sufficient for membrane insertion and crista remodeling. We identified heat shock protein 60 (Hsp60) as the CYP11A1-interacting protein and showed that Hsp60 is required for CYP11A1 accumulation and crista remodeling. Finally, we found that the small MIC10 subcomplex of the mitochondrial contact site and cristae organizing system was reduced when CYP11A1 was overexpressed.

**Conclusions:**

CYP11A1 participates in the formation of tubulovesicular cristae in the mitochondria of steroidogenic cells. Its A’-helix is sufficient for the formation of tubulovesicular cristae and for protein integration into the membrane. CYP11A1 interacts with Hsp60, which is required for CYP11A1 accumulation. The accumulation of CYP11A1 leads to the reduction of MIC10 complex and changes mitochondrial structure.

**Supplementary Information:**

The online version contains supplementary material available at 10.1186/s12929-022-00846-7.

## Background

The first step of steroid production takes place in the mitochondria, in which pregnenolone (P5) is produced from the precursor cholesterol by the enzyme cytochrome P450 11A1 (CYP11A1) [1]. CYP11A1 is a protein in the cytochrome P450 family. It catalyzes the side-chain cleavage of cholesterol, thus this enzyme is also known as cholesterol side-chain cleavage enzyme (abbreviated as P450scc or SCC). This reaction requires electron carriers, adrenodoxin reductase and adrenodoxin, in the inner mitochondrial membrane [2]. Adrenodoxin reductase donates electrons to adrenodoxin. Then adrenodoxin shuttles its electrons to CYP11A1 by shuttling back and forth between ferredoxin reductase and cytochrome P450 [3, 4].

Human CYP11A1 is 521 amino acids (AAs) in length. It bears a 39-AA signal peptide, which is cleaved after CYP11A1 enters the mitochondria. The mature CYP11A1 is composed of 12 helices (helix A to L), four β sheets, and the loops between these structures [5]. CYP11A1 is partially inserted into the inner mitochondrial membrane while the bulk of the protein faces toward the matrix [5]. The regions of CYP11A1 that participate in the interaction with the membrane have been identified to be the hydrophobic A’-helix (AA #57–68) and F-G loop [5–7].

During development, steroidogenic cells differentiate with a gradual increase of steroidogenic enzymes and changes of mitochondrial morphology [8, 9]. CYP11A1 is implicated in vesicle aggregation [10]. In addition, increased CYP11A1 coincides with mitochondrial structural changes such as change of crista shape [8, 9]. For example, spherical mitochondria are present in adrenocortical zona fasciculata and ovarian luteal cells [4, 8], whereas elongated mitochondria are present in testicular Leydig cells [11]. Granulosa cells have elongated mitochondria with lamellar cristae, while luteal cells have spherical mitochondria with tubulovesicular cristae [4, 12].

Mitochondrial cristae are the site of bioenergetics [13]. Crista remodeling in amoeba *Chaos carolinensis* protects the membrane from oxidants and prevents mitochondrial damage [14]. Crista remodeling is also necessary for the complete release of cytochrome c during apoptosis [15]. The integrity of the crista structure is maintained by mitochondria contact site and cristae organizing system (MICOS) [16]. MICOS is divided into two distinct subcomplexes, namely MIC60/MIC19/MIC25 and MIC10/MIC13/MIC26/MIC27 [17]. MIC60 subcomplex is sufficient for cristae junction formation, while MIC10 subcomplex controls lamellar cristae formation [18, 19]. In the absence of MICOS, the localization of the electron complexes is perturbed, thus the cristae structure is impaired [20].

The mitochondria of steroid-producing cells assume special vesicular cristae [8, 9], therefore it is intereresting to find out the mechanism of cristae remodeling during steroidogenic cell differentiation. In the mitochondria of steroidogenic syncytiotrophoblast cell, the amount of dimerized respiratory complex V is reduced by one half [21], indicating the changes in the protein composition during mitochondrial remodeling. But the proteins that shape mitochondrial cristae in these cells are still largely unclear. CYP11A1 can be a candidate protein associated with mitochondrial cristae structure because depletion of CYP11A1 leads to alteration of mitochondrial cristae and loss of tubulovesicular cristae in the adrenocortical cells [22, 23].

In this study, we use COS1 cells because it lacks CYP11A1 and normally cannot synthesize steroids. Yet COS1 has the capacity to be converted into a steroidogenic cell [24], rendering it an ideal cell model for the study. We overexpressed CYP11A1 in COS1 cells to investigate the function of CYP11A1 in mitochondrial cristae remodeling. We found that CYP11A1 remodels mitochondrial cristae from lamellar to tubulovesicular structure and that the A’-helix of CYP11A1 has a role in crista remodeling. Furthermore, we found that Hsp60 regulates CYP11A1 accumulation and mitochondria crista remodeling. Lastly, we found that CYP11A1 remodels cristae via the reduction of MIC10 complex.

## Methods

### Cell culture, transient transfection, generation of stable cell clones

COS1 is a fibroblast-like cell line derived from monkey kidney. HEK 293 T cells are human embryonic kidney cells containing SV40 T-antigen. These cells were grown in DMEM supplemented with 10% FBS and 1% penicillin streptomycin in a 5% CO_2_ incubator at 37 °C. For transfection, 15 µg of plasmids and 45 µL transfection reagent were mixed in 1.5 mL serum-free media before they were added to cells in a 10-cm dish at 60% confluency. Cells were harvested after incubation for 48 h.

We used a tetracycline-inducible (Tet-On) lentiviral system to express exogenous CYP11A1 only when cells were treated with tetracycline or its analog, doxycycline. To generate cell clones for inducible CYP11A1 expression, COS1 cells were co-transduced with two lentiviruses containing *CYP11A1-HF-IRES-EGFP* and *rtTA3* cDNAs in the presence of 8 μg/mL polybrene. Infected cells were selected with 1 μg/mL puromycin for 2 weeks. To isolate cell clones bearing *CYP11A1-HF-IRES-EGFP* cassette, the puromycin-resistant (rtTA3-positive) clonal cells were treated with 1 μg/mL doxycycline and examined for EGFP signal in a fluorescent microscope. EGFP-positive clones were isolated and CYP11A1 expression was further confirmed by immunoblotting with anti-Flag antibody 24 h post-induction with doxycycline. Two independent cell clones, C1 and C4, were selected and used for later experiments.

### Reagents, RNA, plasmids, and cloning

The reagents used here such as antibodies, oligonucleotides, plasmids, enzymes, kits, and software are listed in Additional file 1: Table S1.

For RNA isolation, about 50 mg of 3-month-old zebrafish testis tissue samples were homogenized in 0.5 mL Trizol (Ambion). RNA was extracted by chloroform, precipitated in isopropanol, and dissolved in 50 µL diethyl pyrocarbonate-treated water.

For the cloning of zebrafish *cyp11a1* and *cyp11a2* cDNA, zebrafish testis RNA was used as a template for cDNA synthesis with Maxima Reverse Transcriptase and primers specific for *cyp11a1* and *cyp11a2*. Human *CYP11A1* (AA #1–521) was reported before [25]. *CYP11A1* and all the cDNA fragments (AA #1–39 and AA #1–85) were cloned into the XhoI and EcoRI sites of *pEGFP-N1* vector, or the AflII and BamHI sites of *pcDNA3-EGFP-APEX2* vector. The A’-helix of human CYP11A1 (AA #57–68) was subcloned into the EcoRI and BamHI sites of *pCYP11A1(39)-EGFP* vector, or *pcDNA3-CYP11A1(39)-EGFP-APEX2* vector. The resulting constructs *(pCYP11A1(39)-EGFP, pCYP11A1(39* + *A’)-EGFP, pCYP11A1(85)-EGFP, pCYP11A1(521)-EGFP, pcDNA3-CYP11A1(39)-EGFP-APEX2, pcDNA3-CYP11A1(39* + *A’)-EGFP-APEX2, pcDNA3-CYP11A1(85)-EGFP-APEX2,* and *pcDNA3-CYP11A1(521)-EGFP-APEX2)* were further validated by DNA sequencing.

For the generation of lentiviral construct that expresses CYP11A1, full-length *CYP11A1* cDNA was inserted into the Eco RI and Bam HI sites of a Tet-On lentiviral vector *PL-SIN-5TO-HF-IRES-EGFP* [26], which contains a C-terminal HA-FLAG pPAX2 (HF) tandem tag and an IRES-EGFP cassette driven by five repeats of Tet operators and a mini-CMV promoter. The resulting plasmid, *PL-SIN-5TO-CYP11A1-HF-IRES-EGFP*, was validated by DNA sequencing. The *pTRIPZ-rtTA3* plasmid harbors a coding sequence for the reverse tetracycline-transactivator 3 (rtTA3) and an IRES-puromycin cassette under the control of an EF1a promoter [26]. The *pPAX2* and *pMD2.G* plasmids for lentiviral packaging were purchased from Addgene.

The lentivirus expressing *PL-SIN-5TO-CYP11A1-HF-IRES-EGFP* or *pTRIPZ-rtTA3* were prepared according to a lentivirus-packaging protocol from Addgene. Briefly, 1 μg lentiviral plasmid, 0.75 μg *pPAX2*, and 0.25 μg *pMD2.G* were co-transfected into HEK 293 T cells with 6 µL TransIT-LT1 transfection reagent (Mirus Bio). Lentivirus-containing supernatants were collected 48 h post-transfection.

### Hsp60 knockdown

Hsp60 was knocked down by *si-HSP60* RNA (Dharmacon) following manufacturer’s instructions. Briefly, cells were transfected with 25 nM of *si-HSP60* RNA for 24 h followed by addition of 1 µg/mL doxycycline and incubation for 24 h to induce exogenous CYP11A1 expression. Cells were transfected again with *si-HSP60* RNA and incubated for another 24 h to ensure adequate depletion of Hsp60 before harvesting.

### Immunofluorescence

Cells grown on coverslips inside a 12-well plate were washed with phosphate-buffered saline (PBS) pH 7.4 and fixed with 4% paraformaldehyde in PBS for 20 min at room temperature. Following three PBS washes, cells were permeabilized in 0.2% Triton X-100 (in PBS) for 10 min. After three PBS washes, the coverslip was blocked with 10% normal goat serum in PBS for 1 h at room temperature. The primary antibody was added to the plate after removal of the blocking solution and incubated overnight at 4 °C. After three PBS washes, the coverslips were incubated with secondary antibody (Alexa Fluor 546 Thermo) and 10 µg/mL DAPI for 1 h at room temperature, washed with PBS three times and mounted on a microscope glass slide. Slides were dried in the dark overnight. Images were acquired using a Zeiss LSM710 inverted confocal microscope and processed using ZEN 2011 (Blue edition) software.

### Protein extraction, membrane protein extraction

Proteins were extracted from cells after homogenization in 2 mL lysis buffer (50 mM Tris HCl pH 7.4, 150 mM NaCl, 1 mM EDTA, 1% digitonin, 1 × protease inhibitor) using an electric homogenizer with an adapter pestle. Cell lysates were incubated on ice for 30 min before centrifugation for 17,000×*g* for one min at 4 °C to remove unbroken cells and debris. Proteins in the supernatant were quantified by Bradford Assay and used for succeeding experiments.

For membrane protein extraction, cells were homogenized in 1 mL of 0.1 M Na_2_CO_3_ pH 11.5 using an electric homogenizer with an adapter pestle followed by incubation on ice for 30 min. The homogenates were centrifuged at 435,400×*g* for 1 h using a Beckman rotor TLA 120.1. Pellet fractions were resuspended in alkaline buffer to achieve homogeneity. All fractions were kept in -80 °C until further processing.

### Identification of CYP11A1-interacting proteins

To identify CYP11A1-interacting proteins, about 4 mg of cell lysate from stable clones C1 and C4 in 500 µL TBS (50 mM Tris pH 7.4, 150 mM NaCl) was incubated with 100 µL anti-FLAG M2 or anti-HA affinity bead slurry (Sigma Aldrich) with gentle shaking at 4 °C overnight. After centrifugation at 8200×*g* for 1 min, beads were washed with 500 µL TBS three times. Proteins were eluted by incubating the beads in 200 ng/µL of 3X FLAG (Sigma Aldrich) or 1X HA peptide (Sigma Aldrich) with gentle rotation at 4 °C for 1 h followed by centrifugation at 8200×*g* for 1 min. Samples were separated on SDS-PAGE for Western blot analysis.

### Isolation of mitochondria

Mitochondria were isolated from cells using Mitochondria Isolation Kit for Mammalian Cells (Thermo Scientific). Briefly, about 4 × 10^7^ cells were lysed and cell nuclei removed by centrifugation at 700×*g* for 10 min at 4 °C. Supernatant was collected and centrifuged at 12,000×*g* for 15 min at 4 °C. Mitochondria in the pellet fraction was collected, washed, and centrifuged again at 12,000×*g* for 5 min at 4 °C. Pelleted mitochondria were solubilized in sample buffer (50 mM Bis–Tris pH 7.2, 50 mM NaCl, 10% w/v glycerol, 0.001% Ponceau S).

### Transmission electron microscopy (TEM)

Cells grown in a 12-well cell culture plate lined with Aclar film (Electron Microscopy Sciences) were fixed with 2.5% glutaraldehyde for 20 min and washed with 0.1 M cacodylate buffer pH 7.2. Cells were then post-fixed with 1% osmium for 30 min, washed with distilled water three times, fixed again in 1% uranyl acetate for 30 min, and washed again with distilled water three times. Cells were then sequentially dehydrated in 50%, 70%, 95% and 100% ethanol for 5 min each and washed three times with distilled water. The film was mounted in rubber mold with Epon resin and incubated at 65 °C for 48 h. Resin blocks were cut into 70 nm thickness by a diamond knife on ultramicrotome (Leica EM UC7). Cell slices were placed on mesh grid then post-stained with 4% uranyl acetate for 3 min and lead citrate for 10 min. Ultrathin sections were viewed on Tecnai G2 Spirit TWIN (Thermo) transmission electron microscope operated at 120 kV. Images were acquired via GATAN CCD SC1000 (4008 X 2672 active pixels) camera and processed by Gatan Digital Micrograph software.

### Enzymatic activity by ELISA assay

To examine the activity of CYP11A1, stable clones C1, C4, and the controls were incubated with 12.5 µM 22-hydroxycholesterol overnight. P5 was detected using an ELISA kit (LDN). Briefly, about 50 µL of cell culture media were pipetted into a microwell plate coated with rabbit anti-P5 antibody followed by the addition of 100 µL of P5-horseradish peroxidase (HRP) conjugate working solution. After incubation for 1 h followed by three washes, 150 µL tetramethylbenzidine and hydrogen peroxide substrate were added to the plate and incubated for another 15 min. The reaction was stopped with the addition of 50 µL 1 M sulfuric acid. The amount of P5 was measured by an Emax Precision Microplate Reader (Molecular Devices) and analyzed by Softmax Pro 5.3.

### Mass spectrometry (MS) analysis

Samples obtained from affinity chromatography were analyzed by matrix-assisted laser desorption/ionization-time of flight (MALDI-TOF) MS for protein identification. Both methods of in-gel and in-solution trypsin digestion were used. For in-gel digestion, bands in Coomassie-stained gel were excised, cut into small pieces, and washed twice with 25 mM NH_4_HCO_3_ in 40% methanol while vortexing for 10 min. The samples were dehydrated with 100% acetonitrile before drying in a vacuum centrifuge. For in-solution digestion, about 4 µg proteins were lyophilized in a vacuum centrifuge for 2–3 h.

Protein samples were redissolved in 30 µL distilled water, and reduced by incubation in 5 mM dithiothreitol, 8 M urea, 50 mM ammonium bicarbonate pH 7.8 at 37 °C for 1 h. Samples were then alkylated with 15 mM iodoacetamide for 30 min in the dark at room temperature before digestion with 12.5 ng/μL sequencing grade modified trypsin in 25 mM ammonium bicarbonate containing 10% v/v acetonitrile for 12–16 h at 37 °C. The reaction was stopped through the addition of formic acid to 5% [27]. After salt removal with Millipore C18 Zip-tip, the peptide solution was dried by vacuum centrifugation before dissolution in 30 µL distilled water.

The mass spectra were acquired using two different machines. First spectra were acquired on a Bruker New UltrafleXtremeTM mass spectrometer equipped with an Nd-YAG laser (255 nm) operating at a rate of 200 Hz. One µL of the protein solution was mixed with 1 µL of matrix (10 mg/mL alpha-cyano-4-hydroxycinnamic acid in 50% acetonitrile/0.1% trifluoroacetic acid) directly on a stainless steel MALDI plate and dried. Spectra were recorded in a reflector positive ion mode using an accelerating voltage of 20 kV. The instrument was calibrated with known standards (ovalbumin, serum albumin, myoglobin, cytochrome c, β-lactoglobulin, and Angiotensin 1) to obtain an accuracy of 5 ppm. The mass spectrum was obtained by averaging 2000 laser shots, and the data were processed and analyzed using Flex Analysis software 3.4 (Bruker, Daltonics).

The second MALDI-TOF MS analysis was performed in positive ion mode with delayed extraction (reflection mode) on a Bruker Autoflex III MALDI TOF/TOF mass spectrometer equipped with a 200 Hz SmartBean Laser. About 0.5 μL of the supernatant from the digest was rigorously mixed with 0.5 μL matrix solution (5 mg/ml dihydrobenzoic acid in 0.1%TFA and 30% acetonitrile), and 0.3 μL aliquots of each resulting mixtures were deposited onto the 384/600-μm MTP AnchorChip (Bruker Daltonics). Data were acquired using FlexControl 3.4 and processed by Flex-Analysis 3.4 (Bruker Daltonics). The data were further processed via Biotools 3.2 (Bruker) package accessing the online Mascot server (www.matrixscience.com) to identify corresponding polypeptides against the Swiss-Prot or NCBI database. The parameters for database searches were set as follows: carboxyamidomethylation on cysteine (fixed modification), oxidation of methionine (variable modification), 60 ppm of peptide mass tolerance, 0.7 Da of fragment mass tolerance and 2 missed cleavages. The representative mass spectra are shown in Additional file 1: Fig. S1.

### Polyacrylamide gel electrophoresis and Western blot

Thirty µg of proteins were loaded on 12% SDS polyacrylamide gel before electrophoresis at 90 V for 15 min followed by 130 V for 2.5 h until the protein ladder was visibly separated. For blue native gel electrophoresis (BN-PAGE), about 50 µg mitochondrial protein was loaded in 3–12% Bis–Tris Gel (Novex). Initial run was set at 150 V for 45 min with 200 mL dark Coomassie blue cathode buffer pH 6.8 (50 mM Bis–Tris, 50 mM Tricine, 10 mL 5% G-250 Coomassie blue) and 600 mL anode buffer pH 6.8 (50 mM Bis–Tris, 50 mM Tricine). After the initial run, the cathode buffer was replaced with 200 mL light Coomassie blue buffer pH 6.8 (50 mM Bis–Tris, 50 mM Tricine, 1 mL 5% G-250 Coomassie blue) and voltage was increased to 250 V for 1 h.

Proteins were transferred on PVDF membrane in 1-L transfer buffer (48 mM Tris, 39 mM glycine, 0.037% SDS, 20% methanol) at 80 V for 2 h. For blue native gels, proteins on the blot were additionally fixed with 8% acetic acid for 15 min.

Blots were blocked in 5% milk in PBS-T pH 7.4 (0.1% Tween20 in PBS) followed by incubation with primary antibodies overnight at 4 °C with slow shaking. After three PBS-T washes, secondary antibody was added and incubated for 1.5 h at room temperature with slow shaking followed by three PBS-T washes. The immunoreactive signals were visualized using an enhanced chemiluminescence substrate in a bio-imaging system (UVP Biospectrum 815). The original uncropped gel pictures are shown in Additional file 1: Figs. S2–S9.

### Oxygen consumption measurement

Oxygen consumption rates of cultured cells were measured using a Seahorse XF Cell Mito stress test kit (Agilent). About 40,000 cells were seeded in a 24-well XF24 microplate (Agilent) and grown in DMEM with or without doxycycline for 6 h. After the monolayer cell culture reached about 90% confluency, cell media were replaced with Seahorse XF DMEM media freshly supplemented with 1 mM sodium pyruvate, 2 mM glutamine and 10 mM glucose and incubated at 37 °C without CO_2_ for 1 h before oxygen consumption was measured using an Agilent Seahorse Analyzer. Cells were treated sequentially with 0.5 µM oligomycin, 2 µM carbonyl cyanide-4-trifluoromethoxyphenylhydrazone, and 0.5 µM rotenone/antimycin. Data were analyzed using Wave Software and Seahorse XF Cell Mito Stress Test Report Generator.

### Quantification and statistical analysis

Western blot results were quantified using Image J software. For cristae structure quantification, more than 50 cells were counted from each sample. Data are shown as mean with standard deviation (SD). Unpaired non-parametric *t*-test was used for statistical analysis. N represents the number of independent experiments. For correlating the amounts of CYP11A1 and MIC10 complexes, linear regression was obtained by plotting the data and analyzed using non-linear fit of XY correlation. All statistical analyses were performed using GraphPad Prism8.

## Results

### CYP11A1 remodels mitochondria cristae into tubulovesicular shape

We used COS1 cells in our studies because COS1 cells contain endogenous adrenodoxin and adrenodoxin reductase, two proteins in the electron transport chain for steroid synthesis [24]. Although COS1 cells express no endogenous CYP11A1 and therefore is non-steroidogenic, it can synthesize P5 when it is made to express CYP11A1 [24]. This property makes COS1 an ideal cell model for the investigation of CYP11A1 functions.

**Fig. 1:**
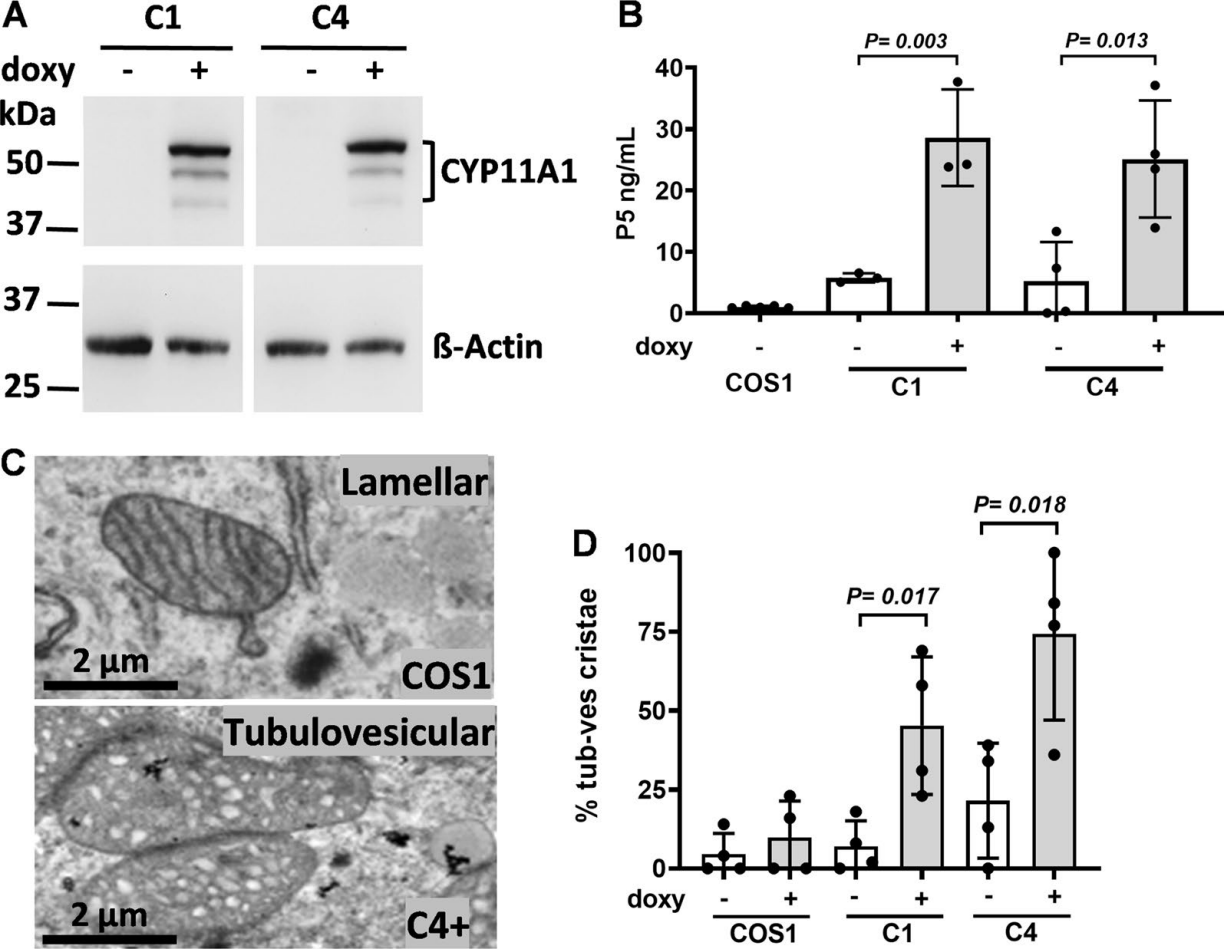
CYP11A1 remodels mitochondria cristae into tubulovesicular shape. **A** Western blot showing CYP11A1 expression in C1 and C4 cells when induced with doxycycline (+ doxy). **B** The amount of P5 production is increased after induction of CYP11A1 with doxycycline in C1 and C4 cells. **C** TEM images of lamellar cristae in COS1 cells and tubulovesicular cristae in stable cell clone C4 after doxycycline induction of CYP11A1 (C4 +). **D** More cells contain tubulovesicular cristae in their mitochondria after induction with doxycycline (doxy) observed under TEM. Unpaired *t*-test was used, and data shown are mean with standard deviation

To study the function of CYP11A1, we engineered a tetracycline-inducible Tet-On system in COS1 cells for the induction of CYP11A1 expression in the presence of tetracycline analog doxycycline. In this system, cells normally did not express exogenous proteins. The expression of CYP11A1 was induced by the addition to doxycycline. Two independent cell clones, C1 and C4, were selected. Immunoblotting assays confirmed that these cells expressed CYP11A1 only in the presence of doxycycline (Fig. 1A).

To test whether the overexpressed CYP11A1 is functional, we incubated the cells with the substrate 22-hydroxycholesterol overnight, and examined the amount of product P5, which is produced using the endogenous electron donors. We detected P5 production only when CYP11A1 was induced by doxycycline in C1 and C4 clones. These experiments indicate that CYP11A1 expressed in these cells is enzymatically active (Fig. 1B).

CYP11A1 is a mitochondrial protein. We therefore examined the structure of mitochondria in normal and CYP11A1-expressed COS1 cells under TEM. Mitochondria in COS1 cells contained lamellar cristae appearing as parallel lines, while those in CYP11A1-expressed cell clones contained tubulovesicular cristae that appear as either elongated or spherical vesicles (Fig. 1C). We counted the number of cells that contain lamellar versus tubulovesicular cristae, and detected higher proportion of cells containing mitochondria with tubulovesicular cristae upon CYP11A1 expression (Fig. 1D). This result indicates that abundant CYP11A1 remodels cristae from lamellar to tubulovesicular.

### Crista remodeling by CYP11A1 is a conserved function in vertebrates

**Fig. 2:**
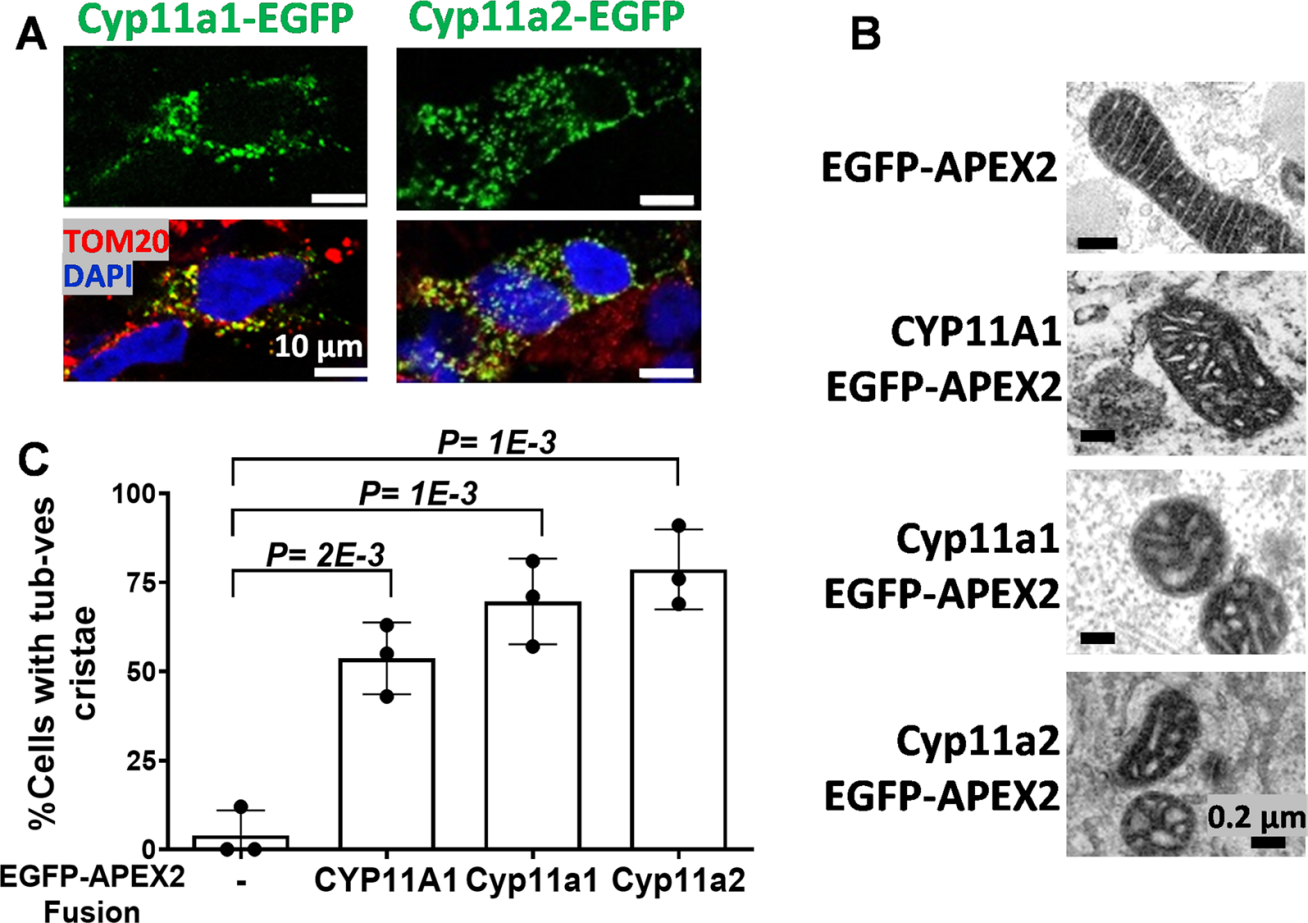
Crista remodeling by CYP11A1 is a conserved function among vertebrates. **A** Immunohistochemistry images of zebrafish Cyp11a1-EGFP and Cyp11a2-EGFP transfected in COS1 cells showing localization of EGFP in mitochondria. TOM20 (red) is a marker for mitochondria, and DAPI (blue) stains the nucleus. **B** TEM images of mitochondria of COS1 cells transfected with human CYP11A1 and zebrafish Cyp11a1 and Cyp11a2 fused to EGFP-APEX. **C** Quantification of tubulovesicular structure observed under TEM. More than 50 cells were counted for quantification of each sample. Unpaired *t*-test was used, and data shown are mean with standard deviation

To check whether the crista remodeling function of CYP11A1 is conserved during evolution, we examined the zebrafish homologues of human CYP11A1. Zebrafish has two such genes, *cyp11a1* and *cyp11a2* [28, 29]. We cloned both cDNAs and placed them into vectors for the expression of proteins fused with EGFP or EGFP-APEX2. The EGFP fusion is for easy immunofluorescence detection; and APEX2 has ascorbate peroxidase activity, which is stained as dark particles under TEM, thus facilitating the detection of exogenous protein [30]. These plasmids were transiently transfected into COS1 cells and the overexpressed proteins were viewed under fluorescence microscopy. Both zebrafish Cyp11a1-EGFP and Cyp11a2-EGFP were present in mitochondria with a punctate pattern and colocalized with a mitochondrial protein, TOM20 (Fig. 2A). Overexpression of zebrafish Cyp11a1-EGFP-APEX2 and Cyp11a2-EGFP-APEX2 also changed mitochondrial cristae to tubulovesicular when examined under TEM analysis (Fig. 2B). In addition, a higher percentage of zebrafish Cyp11a1-EGFP-APEX2 or Cyp11a2-EGFP-APEX2-expressing cells contained tubulovesicular cristae, similar to the human CYP11A1(521)-EGFP-APEX2-expressing cells (Fig. 2C). This result shows that cristae remodeling is a conserved function of CYP11A1 from zebrafish to human species.

### The A’ helix targets CYP11A1 into the membrane and remodels mitochondria cristae

**Fig. 3:**
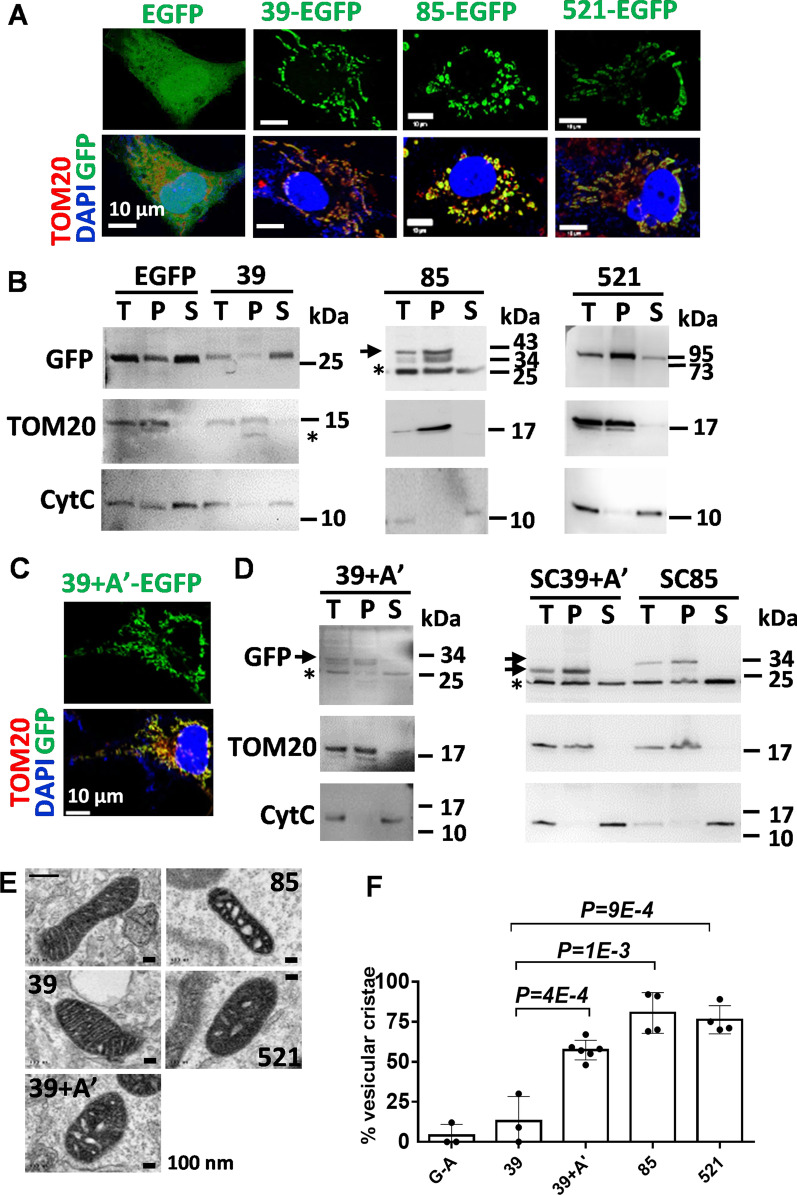
The A’ helix targets CYP11A1 into the membrane and remodels mitochondria cristae. **A** Immunohistochemistry images of EGFP-fused CYP11A1 fragments transfected in COS1 cells. **B** Immunoblotting of EGFP-fused CYP11A1 fragments after alkaline buffer extraction and fractionation by ultracentrifugation. The EGFP and 39-EGFP (abbreviated as 39) proteins were in the supernatant, while the 85- and 521-EGFP proteins were in the pellet. Asterisk indicates non-specific binding of antibody used (T: total; P: pellet; S: supernatant). TOM20 is a control for membrane protein, and cytochrome c (Cyt C) is a control for soluble protein. **C** Immunohistochemistry images of EGFP-fused CYP11A1 signal peptide (AA #1–39) and the anchoring region A’-helix (39 + A’-EGFP) showing its localization in the mitochondria. TOM20 (red) is a marker for mitochondria, and DAPI (blue) stains the nucleus. **D** Partitioning of EGFP-fused CYP11A1 fragments with the membrane-anchoring region A’-helix in transient transfection (39 + A’) or in a stable clone (SC39 + A’) detected by Western blots. The EGFP protein fused to AA #1–85 in a stable clone (SC85) also goes to the pellet. Asterisks indicate non-specific band (T: total; P: pellet; S: supernatant). **E** TEM images of mitochondria of COS1 cells transfected with various fragments of human CYP11A1 fused to EGFP-APEX2. COS1 cells transfected with EGFP-APEX2 only are denoted with a “- “ sign. **F** The A’-helix is sufficient to change cristae into tubulovesicular as quantified after TEM observation. All protein fragments are fused to EGFP-APEX (G-A). More than 50 cells were counted for quantification of each sample. Unpaired *t*-test was used, and data shown are mean with standard deviation

To identify the functional domain of CYP11A1 that remodels mitochondrial cristae, we cloned various fragments of *CYP11A1* cDNA in frame with EGFP-coding sequence. These plasmids were transfected into COS1 cells for overexpression of proteins containing CYP11A1 fragments fused to EGFP. EGFP alone was present evenly in the nucleus and cytoplasm (Fig. 3A). EGFP fused N-terminal fragments (39-EGFP, AA #1–39; 85-EGFP, AA #1–85; 521-EGFP, AA #1–521) of CYP11A1, however, were localized to the mitochondria as shown by the punctate green fluorescence colocalized with mitochondrial marker protein TOM20 (Fig. 3A). These results indicate a mitochondrial targeting function for the N-terminus of CYP11A1.

CYP11A1 is an integral membrane protein. To address whether these CYP11A1 fragments retain the ability to integrate into the mitochondrial membrane, we examined the presence of EGFP-fused CYP11A1 fragments in the soluble or membrane fractions after alkaline extraction. TOM20, a mitochondrial membrane protein, was used as a control for membrane fraction, and cytochrome c as a control for soluble fraction. The first 39 AAs of CYP11A1 is the mitochondrial targeting sequence, which is cleaved after the protein enters the mitochondrion [4]. Although our Western blots always had a nonspecific band located at the position of ~ 25 kDa overlapping with the band of free EGFP protein, we could detect an enhanced intensity of EGFP signal in the supernatant from cells expressing 39-EGFP protein (Fig. 3B). This result is consistent with the notion that the first 39 AAs of CYP11A1 functions as the mitochondrial targeting sequence that is cleaved upon mitochondrial import. The 85- and 521-EGFP were detected only in the pellet fraction, indicating that they are tightly associated with the membrane (Fig. 3B). This finding suggests that AA #39–85 of CYP11A1 may possess a domain that contributes to the integration of CYP11A1 to the mitochondrial membrane.

The crystal structure of CYP11A1 reveals an A’-helix spanning AA #57–68 [5], which is within the candidate region (AA #39–85) for membrane insertion. To test this hypothesis, we fused the A’-helix (AA #57–68) to 39-EGFP to become 39 + A’-EGFP and transiently expressed this protein in COS1 cells. This 39 + A’-EGFP protein, containing the mitochondrial targeting sequence, was localized to the mitochondria as shown by the punctate appearance colocalized with TOM20 upon immunofluorescence detection (Fig. 3C). It was enriched in the membrane fraction (Fig. 3D). To further confirm this, we generated stable COS1 cells stably overexpressing 39 + A’-EGFP (SC39 + A’) and 85-EGFP (SC85). In agreement with the above observation, these A’-helix-containing EGFP fusions were also found in membrane fractions (Fig. 3D). These results indicate that the A’-helix, together with the mitochondrial targeting sequence AA #1–39, is sufficient for inserting the passenger protein into the mitochondrial membrane.

To examine the effect of these CYP11A1 fragments on mitochondrial structure, we used the CYP11A1 fused with EGFP-APEX2 tag for easy detection under TEM (Fig. 3E). While ectopic EGFP-APEX2 (G-A) and 39-EGFP-APEX2 (39-G-A) fusion proteins did not affect the structure of mitochondrial lamellar cristae, mitochondria with tubulovesicular cristae were found in cells with ectopic 39 + A’-, 85-, and 521-G-A fusion proteins (Fig. 3F). These results demonstrated that the A’-helix of CYP11A1 can mediate membrane association and remodel mitochondrial cristae.

### CYP11A1 does not change mitochondria respiration activity

**Fig. 4:**
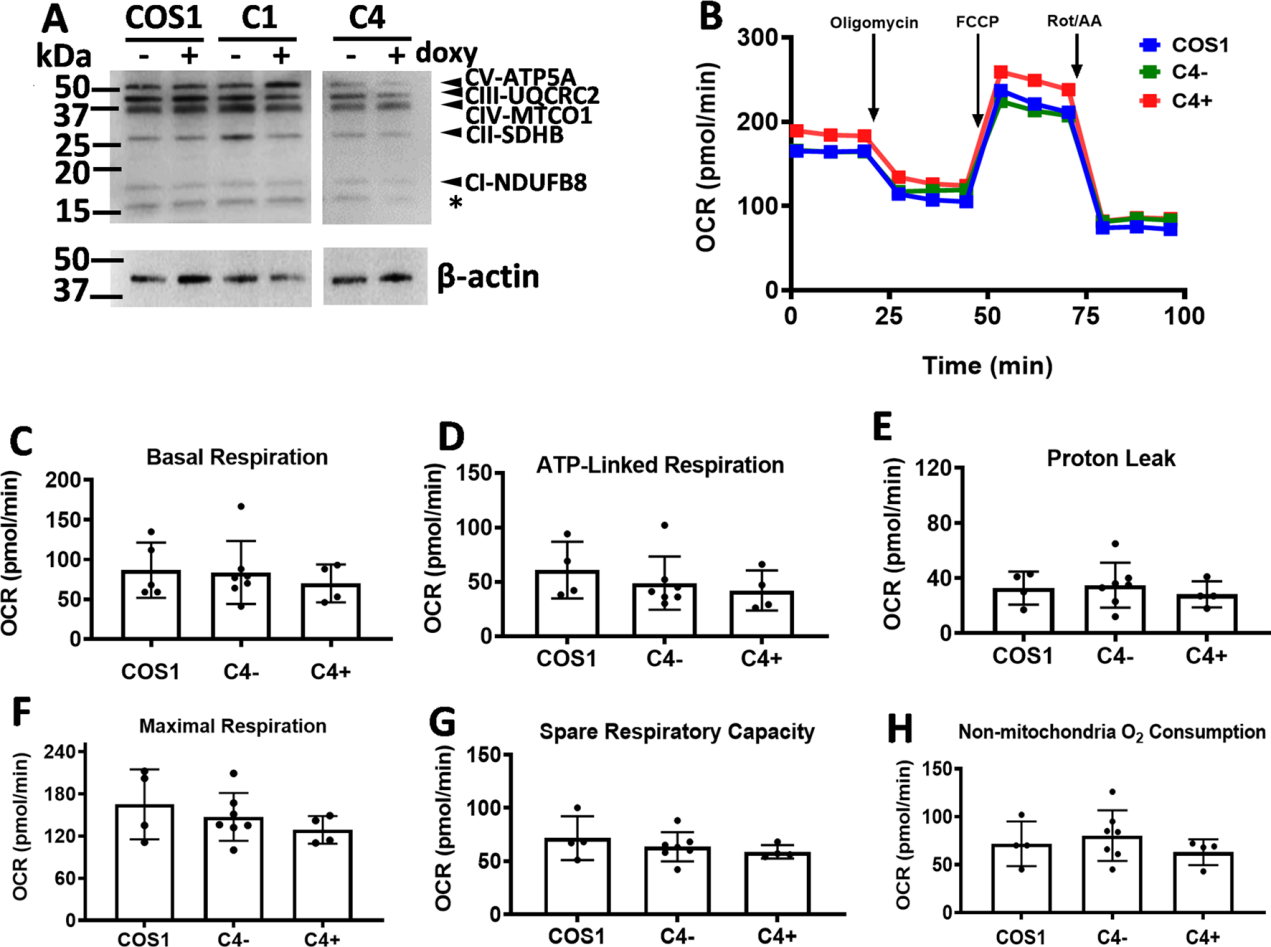
CYP11A1 does not change mitochondrial respiration activity. **A** Western blot showing similar amounts of components of the electron transport complex from stable clones of CYP11A1 (C1, C4) with or without induction of CYP11A1. *Indicates non-specific bands. **B** No difference in overall mitochondria respiration in COS1 and C4 cells with ( +) or without (−) CYP11A1 induction detected by Seahorse Analyzer. **C**–**H** Different parameters of mitochondria respiration in SC C4 show no significant difference among samples with or without CYP11A1

Mitochondria carry out oxidative phosphorylation for energy production. Since CYP11A1 remodels mitochondria cristae from lamellar to tubulo-vesicular structure, we checked if mitochondrial respiration was affected by CYP11A1 overexpression. First, we checked the expression of respiratory complexes in CYP11A1-expressing stable clones C1 and C4 by detecting the protein subunits of each complex as follows: NDUFB8 (complex 1, CI), SDHB (complex II, CII), UQCRC2 (complex III, CIII), MTCO1 (complex IV, CIV), and ATP5A (complex V, CV). Comparable amounts of proteins in CI to CV complexes were detected in cells with or without overexpressed CYP11A1 (Fig. 4A). Analyzing real-time mitochondrial respiration in a Seahorse Analyzer, we found that no significant change of the overall oxygen consumption in C4 cells with or without doxycycline (Fig. 4B). Individual mitochondrial respiration parameters, including basal respiration (Fig. 4C), ATP-linked respiration (Fig. 4D), proton leak (Fig. 4E), maximal respiration (Fig. 4F), spare respiratory capacity (Fig. 4G), and non-mitochondrial oxygen consumption (Fig. 4H) were also not significantly changed upon CYP11A1 overexpression. Furthermore, the formation of respiratory supercomplex (Additional file 1: Fig. S10) and the mitochondrial membrane potential (Additional file 1: Fig. S11) were also unchanged. Therefore, CYP11A1 overexpression does not appear to affect mitochondrial respiration of COS1 cells. Our data are consistent with the report showing that steroidogenic syncytiotrophoblast and their precursor cytotrophoblast have similar ability to use oxygen for ATP production [21].

### Hsp60 together with CYP11A1 induce mitochondria crista remodeling

**Fig. 5:**
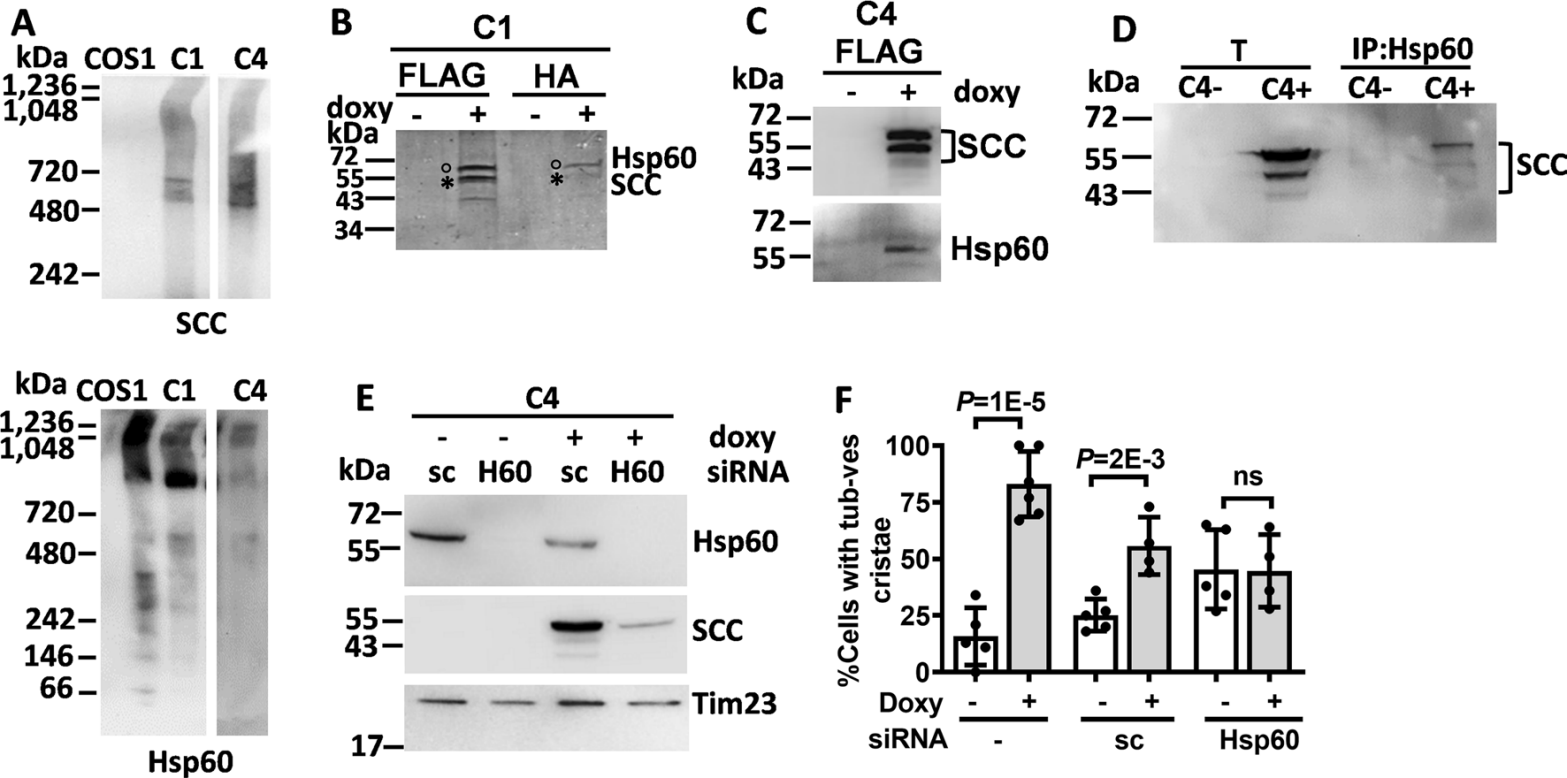
Hsp60 together with CYP11A1 induce mitochondria crista remodeling. **A** Blue native gel followed by immunoblot detection of CYP11A1 (abbreviated as SCC) and Hsp60 isolated from mitochondria of stable clones of CYP11A1. SCC and Hsp60 complexes appear to form at around 480 to 720 kDa and ~ 900 kDa, respectively. **B** Coomassie-stained gel of FLAG and HA immunoprecipitation of stable clone C1. MS analysis indicates that the bottom band is CYP11A1 (SCC, labeled by an asterisk), while the top band is Hsp60 (labeled by a circle). **C** Western blot showing the proteins eluted from the FLAG beads contain CYP11A1 (SCC) and Hsp60 after the induction of C4 cells with doxycycline (doxy). **D** SCC is present after the C4 lysate is induced by doxycycline (+ doxy) and immunoprecipitated by Hsp60 antibody. T: total fraction, IP: Hsp60 immunoprecipitate. **E** Western blots showing the depletion of Hsp60 in C4 cells by Hsp60 siRNA (H60). The amount of CYP11A1 (SCC) is reduced after depletion of Hsp60. Scrambled siRNA (sc) and Tim23 are as negative control and loading control, respectively. **F** Depletion of Hsp60 by siRNA eliminated the induction of mitochondrial tubulovesicular cristae by doxycycline observed under TEM in stable cell clone C4. Unpaired *t*-test was used, and data shown are mean with standard deviation

CYP11A1 often exists in complexes [31]. We examined the size of CYP11A1 complexes by blue native polyacrylamide gel electrophoresis (BN-PAGE), and found that it existed in a few super-complexes between the sizes of 480 to 720 kDa (Fig. 5A). It indicates that CYP11A1 may interact with different proteins forming multiple complexes inside the cell. To search for CYP11A1-associated proteins, we immunoprecipitated CYP11A1 and its associated proteins in the C1 and C4 cell lysate by anti-FLAG or anti-HA beads. The proteins eluted from the FLAG or HA beads were processed with in-solution or in-gel digestion before submission for MS analysis. Eight in-solution digestions were performed for MS analyses, and in all eight experiments we identified Hsp60, gene name *heat shock protein family D member 1* (*HSPD1*) as the major CYP11A1-associated protein (Table 1). In addition, gel analysis of anti-FLAG and anti-HA immunoprecipitates revealed two or three major bands detected by Coomassie blue staining in CYP11A1-expressing cells (Fig. 5B). MS analysis of these protein bands revealed that one prominent band was CYP11A1 and the other band was Hsp60. We therefore used two different immunological methods to confirm Hsp60 as the CYP11A1-interacting protein. Immunoblot analysis of the anti-FLAG immunoprecipitate validated the presence of both CYP11A1 and Hsp60 (Fig. 5C). Reciprocally, immunoprecipitation of cell lysate with anti-Hsp60 antibody followed by immunoblot analysis also detected CYP11A1 in the immunoprecipitate when cells were induced by doxycycline (Fig. 5D). We therefore confirmed an interaction between Hsp60 and CYP11A1. This result is consistent with an earlier report showing the interaction of Hsp60 and CYP11A1 [32].

**Table 1:** CYP11A1 interacting proteins identified by MS

Uniprot ID	Official Gene Symbol	Protein	Mol Wt (Dalton)	Database Search Score	Matched peaks	% Protein coverage
CP11A_HUMAN	CYP11A1	Cholesterol side-chain cleavage enzyme	60,103	4.1E11	43/130	68
CH60_PONAB	HSPD1	60 kDa heat shock protein	60,998	4E3	17/130	34

Proteins in the lysate of doxycycline-induced C1 cells were absorbed to the FLAG beads, eluted, and subjected to MALDI-TOF detection

Mol Wt = molecular weight; matched peaks = number of mass spectrometric peaks that match the proteins in the database/total number of mass spectrometric peaks; % protein coverage = percentage of AAs identified by mass analysis/total number of AAs in the protein

We further analyzed the Hsp60 complexes by BN-PAGE followed by immunoblotting and found that Hsp60 existed in complexes of sizes > 900 kDa (Fig. 5A). This result is consistent with the function of Hsp60 as a molecular chaperon present in large protein complexes [33].

To understand the role of Hsp60-CYP11A1 interaction, we performed si-RNA-mediated depletion of Hsp60 in C4 cells. Scrambled siRNA (sc) was used as a negative control. When Hsp60 was depleted by H60-siRNA, a reduced amount of CYP11A1 was also detected (Fig. 5E). This result indicates that the accumulation of mitochondrial CYP11A1 requires Hsp60. This result is consistent with the earlier finding that Hsp60 promotes progesterone synthesis [32].

We then analyzed mitochondrial morphology by TEM. Consistent with the earlier data, the mitochondria with tubulovesicular cristae were found in a high proportion of CYP11A1-expressing cells in the presence or absence of control sc (Fig. 5F). Depletion of Hsp60, however, completely blunted the effect of CYP11A1 overexpression (Fig. 5F). Therefore, Hsp60 is required for mitochondrial cristae remodeling induced by CYP11A1.

### MICOS10 complex is reduced when CYP11A1 complex is overexpressed

**Fig. 6:**
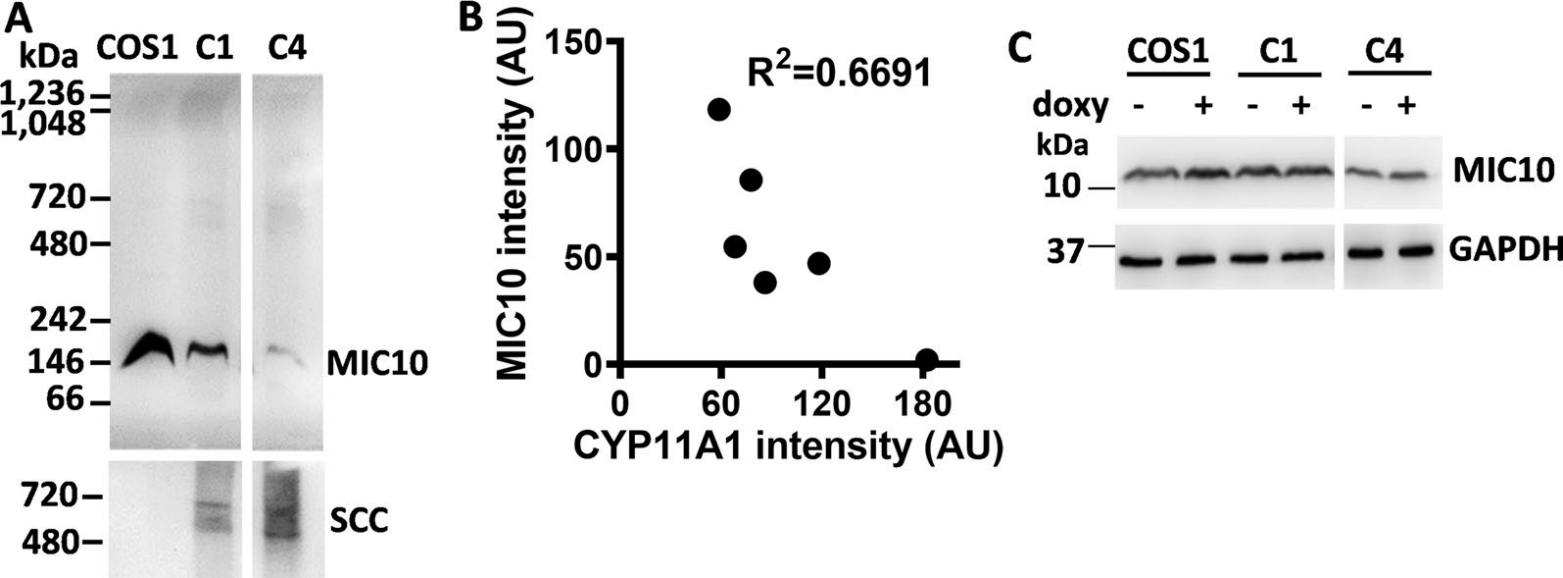
MIC10 subcomplex is reduced when CYP11A1 complex is overexpressed. **A** Immunoblot detection of MIC10 and CYP11A1 complexes after separation of protein complexes from mitochondria of stable clones that overexpress CYP11A1 by blue-native gel electrophoresis. The names of the cells are written on top of each lane. Overexpression of CYP11A1 correlates with reduced MIC10 complex. **B** Quantification of band intensity for MIC10 and CYP11A1 complexes in CYP11A1-expressing C4 cells in arbitrary units (AU). Graph shows inverse relationship of CYP11A1 and MIC10 complexes. **C** Western blot of proteins separated by SDS-PAGE showing unchanged monomeric MIC10 amount in stable clones in the presence ( +) or absence (−) of doxycycline (doxy) induction

To further understand the mechanism underlying CYP11A1-regulated cristae remodeling, we examined proteins in the mitochondrial contact site and cristae organizing system (MICOS), which organizes mitochondrial inner membrane for the formation of mitochondrial cristae [18]. The MICOS complex is divided into MIC60 and MIC10 subcomplexes [17]. BN-PAGE detected a decrease of the amount of ~ 200-kDa MIC10 complex in CYP11A1-expressing C1 and C4 cells (Fig. 6A), but the amount of MIC60 complex was not changed (data not shown). To further confirm this, we quantified the intensities of CYP11A1- and MIC10-immunoreactive complexes in CYP11A1-expressing C4 cells using Image J software and plotted the results of six independent experiments on a graph. Each dot represents the result from one independent experiment. These dots fit a linear regression with a negative correlation between the amount of MIC10-containing complex and that of CYP11A1-containing complex. (Fig. 6B). The expression of MIC10 monomer as examined by SDS-PAGE, however, was unchanged (Fig. 6C). These results indicate that CYP11A1 overexpression does not change the amount of MIC10 monomer, but reduces the formation of MIC10 complex.

## Discussion

In this article we have examined the role of CYP11A1 inside the mitochondrial membrane in addition to its known function in steroid synthesis. We show that the A’-helix of CYP11A1 is sufficient to bring CYP11A1 into the membrane and to change mitochondrial cristae from lamellar to tubulovesicular. We also show that CYP11A1 interacts with Hsp60. CYP11A1 remodels cristae probably by reducing the amount of MIC10 complex inside the mitochondrial cristae junction. We have therefore delineated the participation of Hsp60 and MIC10 in this process.

When a gene function such as the novel role of CYP11A1 is examined, both loss-of-function and gain-of-function tests should be used. CYP11A1 is a marker of steroidogenic cell. Cells devoid of CYP11A1 are no longer steroidogenic, as shown by our previous paper [34]. We have also previously shown that the mitochondria of steroidogenic cells lack discernable cristae when they are devoid of CYP11A1 [22]. Those papers provide loss-of-function evidence for the roles of CYP11A1 in steroidogenesis and in shaping mitochondrial cristae. Now we further investigate the mechanism of this novel role of CYP11A1 using a gain-of-function test. We overexpress CYP11A1 in COS1 cells and show that these cells now produce the first steroid product, P5, and their mitochondrial cristae become tubulovesicular, the same as in steroidogenic cells. Thus, both gain-of-function and loss-of-function tests demonstrate the role of CYP11A1 in regulating the structure of mitochondrial cristae.

The formation of tubulovesicular cristae in steroid-producing cells is in parallel with their increased capacity for steroid production and the increased CYP11A1 expression [4]. This occurs for the differentiation of steroidogenic cells in the adrenal from the granulosa to fasciculata [8], in the ovary from granulosa into luteal cells [12], and in the placenta from cytotrophoblasts to syncytiotrophoblasts [9]. It appears that the formation of vesicular membrane facilitates membrane packing in a small space for efficient protein docking and for diffusion of hydrophobic cholesterol and steroids inside the membrane [21]. It appears that the abundance of CYP11A1 is required for cristae remodeling in vivo although some steroids are already produced when a small amount of CYP11A1 is present.

Steroidogenic cells are characterized by the abundance of CYP11A1, compact vesicular cristae membrane, and ample steroid synthesis; and these three characteristics are tightly associated. CYP11A1 can trigger steroid production by way of its enzymatic activity and its ability to reshaping membranes, resulting in final differentiation and maturation of steroidogenic cells. This step also completes the final step of cell differentiation so that steroidogenic cells are fully equipped with the ability to produce large amounts of steroids when fully differentiated.

Because changes of cristae shapes depend on the perturbation of membranes, the insertion of enough CYP11A1 into the membrane is probably required for adequate membrane. CYP11A1 requires membrane insertion to remodel cristae. CYP11A1 anchors into the inner mitochondrial membrane via two putative anchoring regions, A’-helix and F-G loop [5–7]. Here we show that one anchoring region, the A’-helix, is sufficient to insert CYP11A1 into the inner mitochondria membrane and to remodel cristae. We have not rigorously tested the role of the F-G loop because it appears to be dispensable in our deletion experiment and because in our preliminary tests overexpression of the protein fragments containing the F-G loop leads to protein aggregation. The F-G loop, however, may still possess the membrane anchoring and crista remodeling function similar to the A’-helix under the condition when the A’-helix is made not functional. It is possible that CYP11A1 may possess multiple domains for the same function to ensure proper membrane insertion and remodeling. This strategy of redundancy is often used by the organism when a function is very important for survival. This possibility, however, needs to be tested.

Hsp60 is a chaperone protein that regulates mitochondrial protein folding and assembly [35]. In addition, it promotes progesterone synthesis by binding to cholesterol and interacting with StARD3 and CYP11A1 [32]. Our result showing that the depletion of Hsp60 leads to reduction of CYP11A1 is consistent with the role of Hsp60 as a chaperone. In addition, CYP11A1 relies on Hsp60 to remodel cristae from lamellar to tubulovesicular structure. This is probably because Hsp60 is required for the accumulation of abundant CYP11A1, and the abundance of CYP11A1 is necessary for cristae remodeling.

We noticed that the amount of Hsp60 is reduced when cells overexpress CYP11A1 (Figs. 5A, E). There appears to be a feedback mechanism that adjusts the amount of Hsp60 and CYP11A1 in the cell to reach a balance. The mechanism controlling this feedback inhibition is not clear now and is worth further investigation.

Mitochondrial cristae are regulated by mitochondria contact site and cristae organizing system (MICOS) in the inner mitochondria membrane. Here we show that when CYP11A1 is overexpressed, the amount of MIC10 subcomplex is reduced, although the expression of monomeric MIC10 is unchanged. Thus, the anchorage of CYP11A1 into the inner mitochondria membrane may cause the dissolution or displacement of MIC10 subcomplexes. CYP11A1 may not be the only molecule that can displace MICOS complexes from the membrane. When MICOS is displaced, the cristae will be disturbed. Therefore, any protein that occupies the cristae to displace MICOS probably will change cristae structure. Any protein that inserts into the mitochondrial inner membrane abundantly will probably shape the membrane morphology. One example is the mitochondrial respiratory complex V, which is located in the mitochondrial inner membrane of the placental syncytiotrophoblast cells and changes the membrane into vesicular shape [21].

## Conclusions

Our finding of reduced MIC10 subcomplex in the mitochondrial membrane explains the mechanism of CYP11A1-dependent mitochondrial cristae remodeling. We also show that the A’-helix is sufficient for membrane insertion and crista remodeling, providing further information about the mechanism of mitochondrial crista reorganization by CYP11A1.

## Supplementary Information

**Additional file 1: Table S1.** Key resources used in this study. **Figure S1.** Mass spectra analysis of stable clone C1 with (C1 +) and without CYP11A1 (C1-) after immunoprecipitation (IP). (A) Spectra from mass analysis of proteins eluted peaks corresponding to peptides. (B) Sequences of CYP11A1 and Hsp60. The polypeptides identified by MALDI-TOF are shown in red. **Figure S2.** The original uncropped data of Fig. 1A. Western blot showing stable clones C1 and C4 overexpressing CYP11A1 when induced with doxycycline (doxy). Boxes indicate bands identified by corresponding antibodies. β-actin was used as a loading control. **Figure S3.** The original uncropped data of Fig. 2A. Immunohistochemical images of zebrafish Cyp11a1-EGFP and Cyp11a2-EGFP transfected in COS1 cells showing localization of EGFP in mitochondria. TOM20 (red) is a marker for mitochondria, and DAPI (blue) stains the nucleus. Boxes mark the images shown in Fig. 2A. **Figure S4.** The original uncropped immunofluorescence data of Fig. 3. Immunohistochemistry images of CYP11A1-fragments-EGFP transfected in COS1 cells showing localization of EGFP in mitochondria. TOM20 (red) is a marker for mitochondria, and DAPI (blue) stains the nucleus. Boxes mark the images shown in Fig. 3. **Figure S5.** The original uncropped immunoblot data of Fig. 3B. Partitioning of EGFP-fused CYP11A1 fragments and anchoring region (A’) examined by immunoblotting after alkaline buffer extraction and ultracentrifugation. The EGFP and 39-EGFP (abbreviated as 39) proteins were in the supernatant, while the 85-, and 521-EGFP proteins were in the pellet. Asterisk indicates non-specific binding of antibody used (T: total; P: pellet; S: supernatant). TOM20 is a control for membrane protein, and cytochrome c (Cyt C) is a control for soluble protein. Boxes mark the bands shown in Fig. 3B. **Figure S6.** The original uncropped immunoblot data of Fig. 3D. Partitioning of EGFP-fused CYP11A1 fragments in a stable clone (SC) detected by Western blots. The EGFP protein fused to AA#1–39 plus the anchoring region (SC39 + A’) and AA#1–85 (SC85) also goes to the pellet. The asterisk indicates non-specific bands (T: total; P: pellet; S: supernatant). Boxes mark the bands shown in Fig. 3D. **Figure S7.** The original uncropped immunoblot data of Fig. 4A. Western blot showing similar amounts of components of the electron transport complex (CI-CV) from stable clones of CYP11A1 (C1, C4) with or without induction of CYP11A1. β-actin was used as loading control. Boxes indicate the bands detected by antibody used for each sample. The asterisk indicates non-specific bands. **Figure S8.** The original uncropped immunoblot data of Fig. 5A-E. Verification of CYP11A1 and Hsp60 interaction and the effect of Hsp60 depletion on CYP11A1. Boxes mark the bands shown in Fig. 5. **Figure S9.** The original uncropped immunoblot data of Fig. 6A and C. Inverse relationship of CYP11A1 and Mic10 complexes. Boxes mark the bands shown in Fig. 6. Figure S9. Inverse relationship of CYP11A1 and Mic10 complexes. (A) Western Blot showing MIC10 and SCC complexes after separation of protein complexes from mitochondria of stable clones that overexpress CYP11A1 by blue-native gel electrophoresis. Reduction of Mic10 complex is shown by decreased band intensity with induced CYP11A1 complex. (B) Western blot of proteins separated by SDS-PAGE showing unchanged MIC10 amount in stable clones in the presence ( +) or absence (−) of doxycycline (doxy) induction in stable clones (C1, C2, C4). Asterisk indicates non-specific bands. **Figure S10.** BN-PAGE of electron supercomplexes using stable clone C4. Electron supercomplexes were detected in COS1 and C4 with ( +) and without (-) doxycycline (doxy). Visible bands of electron supercomplexes are labeled with broken lines. **Figure S11.** Measurement of mitochondrial membrane potential in stable clones of CYP11A1. A. Fluorescent images of COS1, C1, and C4 cells after induction of protein overexpression by doxycycline (doxy). GFP (green) shows the presence of CYP11A1 after induction with doxycycline. TMRM staining (red) shows mitochondrial membrane potential. (B). TMRM intensity was quantified and compared.

## Data Availability

Not applicable.

## Abbreviations

CYP11A1: Cytochrome P450 11A1
Hsp60: Heat shock protein 60
MALDI: Matrix-assisted laser desorption/ionization
TOF: Time of flight
MICOS: Mitochondria contact site and cristae organizing system
MS: Mass spectrometry
P5: Pregnenolone
SCC: Side chain cleavage enzyme
sc: Scrambled siRNA
TEM: Transmission electron microscopy
AA: Amino acid
