# Supplementary material for: The A’-helix of CYP11A1 remodels mitochondrial cristae
Source: J Biomed Sci. 2022 Aug 18;29:61. doi: 10.1186/s12929-022-00846-7 (PMC9386925; doi:10.1186/s12929-022-00846-7)
Supplement: Supplementary file 1 — Additional file 1: Table S1. Key resources used in this study. Figure S1. Mass spectra analysis of stable clone C1 with (C1 +) and without CYP11A1 (C1-) after immunoprecipitation (IP). (A) Spectra from mass analysis of proteins eluted peaks corresponding to peptides. (B) Sequences of CYP11A1 and Hsp60. The polypeptides identified by MALDI-TOF are shown in red. Figure S2. The original uncropped data of Fig. 1A. Western blot showing stable clones C1 and C4 overexpressing CYP11A1 when induced with doxycycline (doxy). Boxes indicate bands identified by corresponding antibodies. β-actin was used as a loading control. Figure S3. The original uncropped data of Fig. 2A. Immunohistochemical images of zebrafish Cyp11a1-EGFP and Cyp11a2-EGFP transfected in COS1 cells showing localization of EGFP in mitochondria. TOM20 (red) is a marker for mitochondria, and DAPI (blue) stains the nucleus. Boxes mark the images shown in Fig. 2A. Figure S4. The original uncropped immunofluorescence data of Fig. 3. Immunohistochemistry images of CYP11A1-fragments-EGFP transfected in COS1 cells showing localization of EGFP in mitochondria. TOM20 (red) is a marker for mitochondria, and DAPI (blue) stains the nucleus. Boxes mark the images shown in Fig. 3. Figure S5. The original uncropped immunoblot data of Fig. 3B. Partitioning of EGFP-fused CYP11A1 fragments and anchoring region (A’) examined by immunoblotting after alkaline buffer extraction and ultracentrifugation. The EGFP and 39-EGFP (abbreviated as 39) proteins were in the supernatant, while the 85-, and 521-EGFP proteins were in the pellet. Asterisk indicates non-specific binding of antibody used (T: total; P: pellet; S: supernatant). TOM20 is a control for membrane protein, and cytochrome c (Cyt C) is a control for soluble protein. Boxes mark the bands shown in Fig. 3B. Figure S6. The original uncropped immunoblot data of Fig. 3D. Partitioning of EGFP-fused CYP11A1 fragments in a stable clone (SC) detected by Western blots. The EGFP protei [file 12929_2022_846_MOESM1_ESM.docx]

**Additional file 1**

**The A’-helix of CYP11A1 remodels mitochondrial cristae**

Karen G. Rosal^1,2^, Wei-Yi Chen^3^, and Bon-chu Chung^1,2,4,*^

^1^ Molecular Cell Biology, Taiwan International Graduate Program, Academia Sinica and Graduate Institute of Life Science, National Defense Medical Center, Taipei, 115, Taiwan

^2^ Institute of Molecular Biology, Academia Sinica, Taipei, 115, Taiwan

^3^ Institute of Biochemistry and Molecular Biology, National Yang Ming Chiao Tung University, Taipei, 112, Taiwan

^4^ Graduate Institute of Biomedical Sciences, Neuroscience and Brain Disease Center, China Medical University, Taichung, 404, Taiwan

^*^ Corresponding Author: Bon-chu Chung, Institute of Molecular Biology, Academia Sinica, Taipei, 115, Taiwan

This Additional file 1 contains one additional table, one additional method, and 11 additional figures.

**Table S1. Key resources used in this study.**

| **Reagent or resource** | **Source** | **Identifier** | **Note** |
| --- | --- | --- | --- |
| **Antibodies** | | | |
| Rabbit anti-CYP11A1 (SCC) | Our own production, see *Biochem J*. 1991;274:813-817 | N/A | 1:10000 |
| Mouse monoclonal anti-cytochrome c | BD Biosciences | 556433 | 1:1000 |
| rabbit polyclonal anti-DYKDDDDK Tag | Proteintech | 20543-1-AP | 1:5000 |
| Mouse monoclonal anti-FLAG M2 | Sigma Aldrich | F3165 | 1:5000 |
| Mouse monoclonal anti-GFP | Proteintech | 66002-1-Ig | 1:5000 |
| Mouse monoclonal anti-Hsp60 (HSP60 H-1) | Santa Cruz Biotechnology | sc-13115 | 1:200 |
| Goat peroxidase-conjugated anti-mouse IgG | Chemicon International | AP124P | 1:5000 |
| Mouse monoclonal anti-Tim23 | Santa Cruz Biotechnology | sc-514463 | 1:1000 |
| Mouse monoclonal anti-OXPHOS for SDS-PAGE | Abcam | ab110413 | 6 µg/mL |
| Mouse monoclonal anti-Minos1 (MIC10) | Novus Biologicals | NBP2-45550 | 1:1000 |
| Rabbit polyclonal anti-TOM20 | Santa Cruz Biotechnology | sc-11415 | 1:2000 |
| Anti-rabbit IgG AlexaFluor 546 | Invitrogen | A10040 | 1:500 |
| Goat peroxidase-conjugated anti-rabbit IgG | Abcam | ab131366 | 1:5000 |
| Goat peroxidase-conjugated anti-mouse IgG | Chemicon International | AP124P | 1:5000 |

| **Reagent or resource** | **Source** | **Note** |
| --- | --- | --- |
| Primer: A’-  5' CGAATTCTGAATGG CTGGCTAAACCTGTACC ATTTCTGGAGGGAGCGGGATCCA 3' | Integrated DNA Technologies | For subcloning A’-helix |
| z_cyp11a1F-EGFP  5’ AGATCTCGAGATGGCCCGCTGGAATGTG 3’ | Genomics Biosci and Tech | For cloning zebrafish Cyp11a1 into pEGFP vector |
| z_cyp11a1R-EGFP  5’ TGCAGAATTCGTCTGCTGGCATTCA 3’ | Genomics Biosci and Tech | For cloning zebrafish Cyp11a1 into pEGFP vector |
| z_cyp11a2F-EGFP  5’ AGATCTCGAGATGGCCCGCTGGAGTCTG 3’ | Genomics Biosci and Tech | For cloning zebrafish Cyp11a2 into pEGFP vector |
| z_cyp11a2R-EGFP  5’ TGCAGAATTCGTCTGCTGGAGTTCA 3’ | Genomics Biosci and Tech | For cloning zebrafish Cyp11a2 into pEGFP vector |
| a1APEX_BsptI_FP  5’ CTTAAGATGGCCCGCTGGAATGTGACGTTC 3’ | Genomics Biosci and Tech | For cloning zebrafish Cyp11a1 into pcDNA3.1-EGFP-APEX2 vector |
| a1APEX_BamHI_RP  5’ GGATCCTCTGCTGGCATTCAGTGG | Genomics Biosci and Tech | For cloning zebrafish Cyp11a1 into pcDNA3.1-EGFP-APEX2 vector |
| a2APEXAflIIFPnewer  5’ TAAACTTAAGATGGCCCGCTGGAGT 3’ | Genomics Biosci and Tech | For cloning zebrafish Cyp11a2 into pcDNA3.1-EGFP-APEX2 vector |
| a2APEXBamHIRPnewer  5’ CCCCGGATCCTCTGCTGGAGTTCAG 3’ | Genomics Biosci and Tech | For cloning zebrafish Cyp11a2 into pcDNA3.1-EGFP-APEX2 vector |

| **Plasmids** | | | |
| --- | --- | --- | --- |
| **Reagent or resource** | **Source** | **Identifier** | **Note** |
| pEGFP-N1 | Clontech | 6085-1 |  |
| pCYP11A1(521)-EGFP | This paper | N/A | plasmid consisting of the full length human CYP11A1 inserted at the XhoI and EcoRI sites of pEGFP-N1 fused with EGFP. |
| pCYP11A1(39)-EGFP | This paper | N/A | plasmid consisting of the human CYP11A1 first 39 amino acids inserted at the XhoI and EcoRI sites of pEGFP-N1 fused with EGFP. |
| pCYP11A1(85)-EGFP | This paper | N/A | plasmid consisting of the human CYP11A1 first 85 amino acids inserted at the XhoI and EcoRI sites of pEGFP-N1 fused with EGFP. |
| pCYP11A1(39+A’)-EGFP | This paper | N/A | plasmid consisting of the human CYP11A1 first 39 amino acids plus the anchoring region of CYP11A1 A’- helix inserted into the EcoRI and BamHI sites of pEGFP-N1 fused with EGFP. |
| pCyp11a1-EGFP | This paper | N/A | plasmid consisting of the zebrafish Cyp11a1 inserted at the XhoI and EcoRI sites of pEGFP-N1 fused with EGFP. |
| pCyp11a2-EGFP | This paper | N/A | plasmid consisting of the zebrafish Cyp11a2 inserted at the XhoI and EcoRI sites of pEGFP-N1 fused with EGFP. |
| pcDNA3-EGFP-APEX2 | This paper | N/A | Plasmid generated by deleting connexin43 from pcDNA3 Connexin43-GFP-APEX2 (Addgene Cat #49385) |
| pcDNA3-CYP11A1(39)-EGFP-APEX2 | This paper | N/A | plasmid consisting of the human CYP11A1 first 39 amino acids inserted into the AflII and BamHI sites of pcDNA3-EGFP-APEX2. |
| pcDNA3.1-CYP11A1(85)-EGFP-APEX2 | This paper | N/A | plasmid consisting of the human CYP11A1 first 85 amino acids inserted into the AflII and BamHI sites of pcDNA3-EGFP-APEX2. |
| pcDNA3-CYP11A1(39+A’)-EGFP-APEX2 | This paper | N/A | plasmid consisting of the human CYP11A1 first 39 amino acids plus the A’-helix inserted into the EcoRI and BamHI sites of pcDNA3-EGFP-APEX2. |
| pcDNA3-CYP11A1(521)-EGFP-APEX2 | This paper | N/A | plasmid consisting of the full length human CYP11A1 inserted into the AflII and BamHI sites of pcDNA3-EGFP-APEX2. |
| pcDNA3-Cyp11a1-EGFP-APEX2 | This paper | N/A | plasmid consisting of the zebrafish Cyp11a1 inserted at the AflII and BamHI sites of pcDNA3-EGFP-APEX2. |
| pcDNA3-Cyp11a2-EGFP-APEX2 | This paper | N/A | plasmid consisting of the zebrafish Cyp11a2 inserted at the AflII and BamHI sites of pcDNA3-EGFP-APEX2 |
| pPAX2 | Addgene | 12260 |  |
| pMD2.G | Addgene | 12259 |  |
| PL-SIN-5TO-CYP11A1-HF-IRES-EGFP | This paper | N/A | The C-terminal HA-FLAG tagged human *CYP11A1* and *EGFP* cDNA inserted into the EcoRI and BamHI sites of *PL-SIN-5TO- HF-IRES-EGFP*. |
| pTRIPZ-rtTA3 | *Mol Cell* 2019;74:268-283 | N/A |  |

| **Softwares** | **Source** |
| --- | --- |
| ImageJ/Fiji | https://imagej.net/Fiji |
| ZEN 2011 (Blue edition) | Carl Zeiss |
| Gatan Digital Micrograph | https://www.gatan.com |
| Softmax Pro 5.3 | Molecular Devices |
| Flex Analysis Software 3.4 | Bruker, Daltonics |
| Wave Software | Agilent |
| GraphPad Prism Version 8.1.1 | GraphPad Software, Inc. |
| MetaXpress Version 6.5.5 | Molecular Devices |

| **Other reagents and kits** | | | |
| --- | --- | --- | --- |
| **Reagent or resource** | **Source** | **Identifier** | **Note** |
| NativePAGE Novex Bis-Tris Gel System | Life Technologies | BN2011BX10 | For native gel electrophoresis |
| Pregnenolone ELISA | Labor Diagnostika Nord | FR E-2700 | For direct quantitative determination of pregnenolone by enzyme immunoassay |
| Mirus TransIT LT1 | Mirus Bio | MIR 2300 | For transient transfection of COS1 cells |
| Maxima Reverse Transcriptase | Thermo Scientific | EP0741 | For the synthesis of zebrafish *cyp11a1* and *cyp11a2* cDNA |
| Mitochondria Isolation Kit for mammalian cells | Thermo Scientific | 89874 | For mitochondria protein extraction from COS1 cells |
| Seahorse XF Cell Mito Stress Test Kit | Agilent Technologies | 103015-100 | For measuring oxygen consumption rate of live cells in real time |
| *si-HSP60* RNA | Dharmacon | SO-2994873G | For Hsp60 (HSPD1) knockdown |
| Trizol | Ambion | 15596026 | For RNA extraction from tissue samples |
| 3X FLAG peptide | Sigma Aldrich | F4799 | For elution of FLAG-tagged protein |
| 1X HA peptide | Sigma Aldrich | 12149 | For elution of HA-tagged protein |
| COS1 | ATCC | CRL-1650™ | Cell line used for all cell culture experiments |
| HEK-293T | ATCC | CRL-3216™ | Cell line used for the generation of lentivirus in the Tet-On system. |
| Aclar film | Electron Microscopy Sciences | 50425-25 | For preparation of cell culture samples for electron microscopy |

**Additional Method**

**Measurement of mitochondria potential**

For measurement of mitochondria potential using stable clones of CYP11A1, a cell-permeable dye, tetramethylrhodamine methyl ester (TMRM, Thermo Fisher), that stains mitochondrial membrane was used. Briefly, about 40,000 cells were grown with DMEM in a 96-well cell culture plate with and without the addition of doxycycline (1 µg/mL). After reaching about 90% confluency, the cell growth medium was replaced with 100 µL TMRM dye (100 µM in DMSO) and incubated for 30 minutes at 37°C with CO_2_. Cells were then washed with PBS. Cells were observed under high content microscopy ImageXpress Micro XL (Molecular Devices) using TRITC/RFP settings and quantified using MetaXpress Version 6.5.5 software. The experiment was repeated thrice.

**Figure S1. Mass spectra analysis of stable clone C1 with (C1+) and without CYP11A1 (C1-) after immunoprecipitation (IP)**. (A) Spectra from mass analysis of proteins eluted peaks corresponding to peptides. (B) Sequences of CYP11A1 and Hsp60. The polypeptides identified by MALDI-TOF are shown in red.

**Figure S2. The original uncropped data of Fig. 1A. Western blot showing stable clones C1 and C4 overexpressing CYP11A1 when induced with doxycycline (doxy).** Boxes indicate bands identified by corresponding antibodies. β-actin was used as a loading control.

**Figure S3. The original uncropped data of Fig. 2A. Immunohistochemical images of zebrafish Cyp11a1-EGFP and Cyp11a2-EGFP transfected in COS1 cells showing localization of EGFP in mitochondria.** TOM20 (red) is a marker for mitochondria, and DAPI (blue) stains the nucleus. Boxes mark the images shown in Fig. 2A.

**
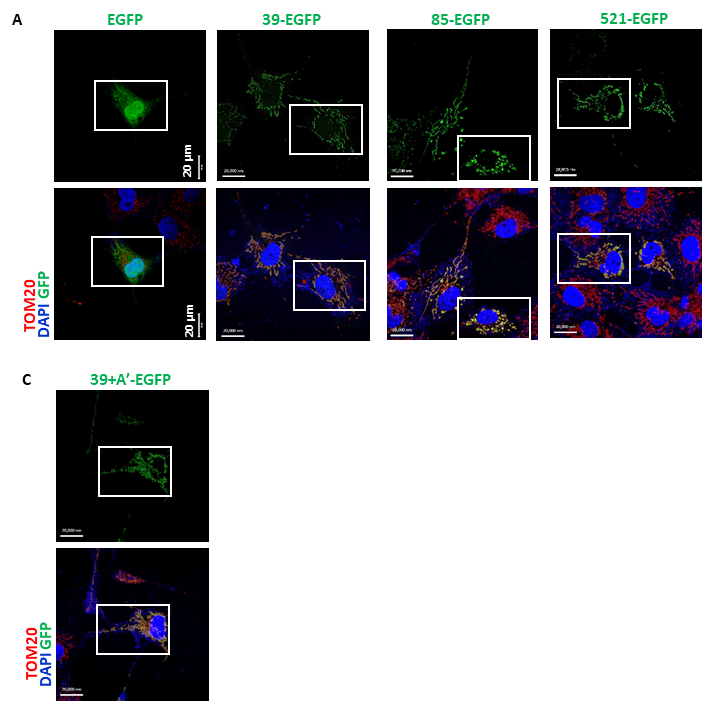
**

**Figure S4. The original uncropped immunofluorescence data of Fig. 3. Immunohistochemistry images of CYP11A1-fragments-EGFP transfected in COS1 cells showing localization of EGFP in mitochondria.** TOM20 (red) is a marker for mitochondria, and DAPI (blue) stains the nucleus. Boxes mark the images shown in Fig. 3.

**Figure S5. The original uncropped immunoblot data of Fig. 3B. Partitioning of EGFP-fused CYP11A1 fragments and anchoring region (A’) examined by immunoblotting after alkaline buffer extraction and ultracentrifugation.** The EGFP and 39-EGFP (abbreviated as 39) proteins were in the supernatant, while the 85-, and 521-EGFP proteins were in the pellet. Asterisk indicates non-specific binding of antibody used (T: total; P: pellet; S: supernatant). TOM20 is a control for membrane protein, and cytochrome c (Cyt C) is a control for soluble protein. Boxes mark the bands shown in Fig. 3B.

**Figure S6. The original uncropped immunoblot data of Fig. 3D. Partitioning of EGFP-fused CYP11A1 fragments in a stable clone (SC) detected by Western blots.** The EGFP protein fused to AA#1-39 plus the anchoring region (SC39+A’) and AA#1-85 (SC85) also goes to the pellet. The asterisk indicates non-specific bands (T: total; P: pellet; S: supernatant). Boxes mark the bands shown in Fig. 3D.

**Figure S7. The original uncropped immunoblot data of Fig. 4A. Western blot showing similar amounts of components of the electron transport complex (CI-CV) from stable clones of CYP11A1 (C1, C4) with or without induction of CYP11A1.** β-actin was used as loading control. Boxes indicate the bands detected by antibody used for each sample. The asterisk indicates non-specific bands.

**Figure S8. The original uncropped immunoblot data of Fig. 5A-5E. Verification of CYP11A1 and Hsp60 interaction and the effect of Hsp60 depletion on CYP11A1.** Boxes mark the bands shown in Fig. 5.

**Figure S9. The original uncropped immunoblot data of Fig. 6A and 6C. Inverse relationship of CYP11A1 and Mic10 complexes.** Boxes mark the bands shown in Fig. 6.

**Supplementary Figure S9. Inverse relationship of CYP11A1 and Mic10 complexes.** (A) Western Blot showing MIC10 and SCC complexes after separation of protein complexes from mitochondria of stable clones that overexpress CYP11A1 by blue-native gel electrophoresis. Reduction of Mic10 complex is shown by decreased band intensity with induced CYP11A1 complex. (B) Western blot of proteins separated by SDS-PAGE showing unchanged MIC10 amount in stable clones in the presence (+) or absence (-) of doxycycline (doxy) induction in stable clones (C1, C2, C4). Asterisk indicates non-specific bands.

**Figure S10.** **BN-PAGE of electron supercomplexes using stable clone C4.** Electron supercomplexes were detected in COS1 and C4 with (+) and without (-) doxycycline (doxy). Visible bands of electron supercomplexes are labeled with broken lines.

**Figure S11. Measurement of mitochondrial membrane potential in stable clones of CYP11A1.** A. Fluorescent images of COS1, C1, and C4 cells after induction of protein overexpression by doxycycline (doxy). GFP (green) shows the presence of CYP11A1 after induction with doxycycline. TMRM staining (red) shows mitochondrial membrane potential. (B). TMRM intensity was quantified and compared.
